# Nanostructured Porous Silicon: The Winding Road from Photonics to Cell Scaffolds – A Review

**DOI:** 10.3389/fbioe.2015.00060

**Published:** 2015-05-11

**Authors:** Jacobo Hernández-Montelongo, Alvaro Muñoz-Noval, Josefa Predestinación García-Ruíz, Vicente Torres-Costa, Raul J. Martín-Palma, Miguel Manso-Silván

**Affiliations:** ^1^Departamento de Física Aplicada, Instituto de Física Gleb Wataghin, Universidade Estadual de Campinas, Campinas, Brazil; ^2^Departamento de Física Aplicada, Universidad Autónoma de Madrid, Madrid, Spain; ^3^Instituto de Ciencia de Materiales de Madrid-CSIC, Spanish CRG Beamline at ESRF, Grenoble, France; ^4^Departamento de Biología Molecular, Universidad Autónoma de Madrid, Madrid, Spain; ^5^Centro de Micro-Análisis de Materiales, Universidad Autónoma de Madrid, Madrid, Spain; ^6^Instituto Nicolás Cabrera, Universidad Autónoma de Madrid, Madrid, Spain

**Keywords:** porous silicon, nanostructure, photonics, optical properties, biomaterial, cell culture, cell scaffold, review

## Abstract

For over 20 years, nanostructured porous silicon (nanoPS) has found a vast number of applications in the broad fields of photonics and optoelectronics, triggered by the discovery of its photoluminescent behavior in 1990. Besides, its biocompatibility, biodegradability, and bioresorbability make porous silicon (PSi) an appealing biomaterial. These properties are largely a consequence of its particular susceptibility to oxidation, leading to the formation of silicon oxide, which is readily dissolved by body fluids. This paper reviews the evolution of the applications of PSi and nanoPS from photonics through biophotonics, to their use as cell scaffolds, whether as an implantable substitute biomaterial, mainly for bony and ophthalmological tissues, or as an *in vitro* cell conditioning support, especially for pluripotent cells. For any of these applications, PSi/nanoPS can be used directly after synthesis from Si wafers, upon appropriate surface modification processes, or as a composite biomaterial. Unedited studies of fluorescently active PSi structures for cell culture are brought to evidence the margin for new developments.

## Introduction

According to Williams (1999), tissue engineering “is the persuasion of the body to heal itself through the delivery to appropriate sites of molecular signals, cells, and supporting structures.” In that sense, a cell scaffold for tissue engineering applications can be defined as a substrate designed to support the appropriate cellular activity, including the facilitation of molecular and mechanical signaling systems, in order to optimize tissue regeneration, without eliciting any undesirable local or systemic responses in the eventual host (Williams, 2008).

As a consequence of its appropriate biomedical properties, porous silicon (PSi) and nanostructured porous silicon (nanoPS) have found increasing applications beyond traditional uses in the field of photonics to the field of tissue engineering as cell scaffold given that its morphology at the micro- and nano-scales can be used to regulate cell behavior (Sun et al., 2007a). Its flexible surface chemistry can be tailored to improve the PSi/nanoPS-cell interfacial properties and thus their interaction (Sun et al., 2007b). Besides, PSi/nanoPS has an advantage over other biomaterials, namely, their intrinsic ability to be easily degraded in aqueous solutions into non-toxic silicic acid (Low et al., 2006). Relevantly, this degradation makes of PSi/nanoPS bioactive materials in simulated plasma, since their corrosion with release of Si(OH)_4_ stimulates calcification and subsequent hydroxyapatite formation (Coffer et al., 2005).

In that sense, research on the use of PSi/nanoPS in the area of biomedicine is increasing given that it is a suitable biomaterial for the industry. Silicon – the substrate generally used to produce it – is a low-cost commodity compatible with high-tech electronic industry. Only in the semiconductor industry, the most relevant market for silicon, it has been predicted an increase from US$125 billion in 1998 to US$ 3.3 trillion in 2020 (Williams, 2003).

Previous reviews of PSi and nanoPS include their use as optical sensors (and particularly biosensors) (Dhanekar and Jain, 2013), as drug-delivery systems (Anglin et al., 2008), as well as their general applications in the biomedical field, including tissue engineering (Martin-Palma et al., 2010). The general spanning field of a nanostructured material derived from a reference technological compound as Si, as well as the recent advances in its use in tissue engineering calls for a revision and categorization of its most relevant applications that could help in focusing new technological applications.

## Nanostructured Porous Silicon: Photonics and Optoelectronics

**Figure 1:**
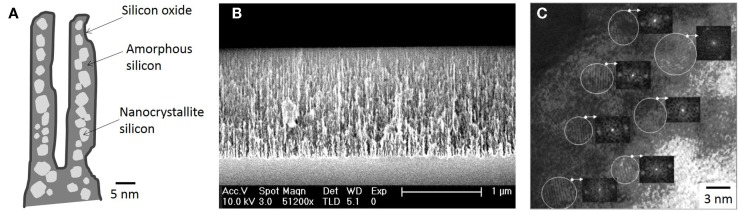
**(A)** Schematic view of the typical distribution of PSi components, **(B)** a cross-sectional FESEM image of a characteristic nanoPS layer, and **(C)** a HRTEM image of nanoPS [**(C)** is reprinted from Martín-Palma et al. (2002)].

Porous silicon was discovered in 1956 by Uhlir (1956) when carrying out electropolishing experiments. However, this milestone was just reported as a technical note, with no particular technological application at that time. Since then, PSi had very few applications until 1990 when its luminescence in the visible wavelength regime at room temperature was discovered by Canham (1990). This effect was attributed to quantum confinement effects given that nanoPS is constituted by silicon nanocrystallites embedded in a porous silica skeleton, as shown in Figure 1A (Bisi et al., 2000). The silicon nanocrystallites are covered by amorphous silicon, which oxidizes over time (Petrova et al., 2000) upon exposure to the atmosphere. This relatively complex porous structure may reach a very large internal surface area, up to ~500 m^2^/cm^3^ according to some authors (Granitzer and Rumpf, 2010), depending on the fabrication parameters. In Figure 1B, a cross-sectional FESEM image of a characteristic columnar PSi layer is shown. In the TEM image of Figure 1C, the individual silicon nanocrystallites, which constitute nanoPS, can be easily identified. These show a spherical shape with typical dimensions in the range between 20 and 80 Å, without a preferential orientation (polycrystalline diffraction pattern) (Martín-Palma et al., 2002).

**Figure 2:**
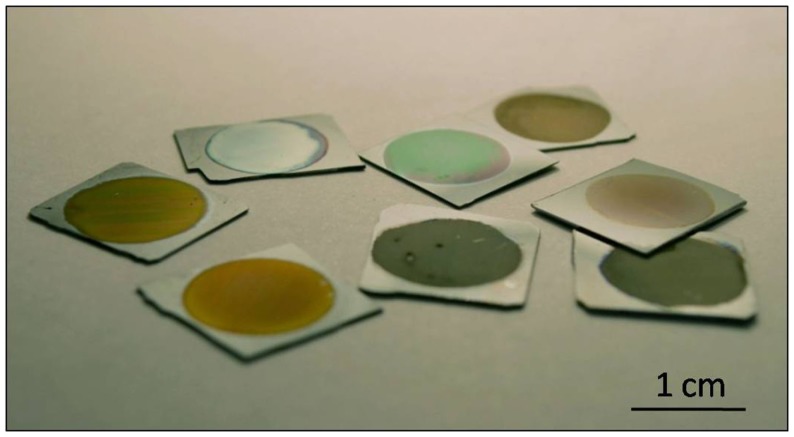
**Image of various PSi samples prepared at different synthesis conditions**.

This particular nanostructure generates photoluminescence and electroluminescence at room temperature in the visible (blue to red) and infrared (Canham, 1990; Halimaoui et al., 1991). The most accepted theories indicate that the blue band can be linked to the presence of silicon dioxide on the surface, while the red band has its origin in quantum confinement effects originated in the silicon nanocrystallites, possibly supplemented by surface states, and the infrared band is correlated with dangling bonds and bandgap luminescence in larger crystallites (Fauchet, 1996; Bisi et al., 2000). These properties made nanoPS a very promising materials for applications in the fields of optoelectronics and photonics, in so much that publications related to nanoPS/PSi grew exponentially in 1990s (Parkhutik, 2000). In fact, to date, most applications of PSi are related to its tunable optical properties, since the refractive index of this material can be varied continuously between the indices of bulk silicon and air by changing the porosity (Torres-Costa and Martin-Palma, 2010). As an example, Figure 2 portrays how the reflectance spectrum from nanoPS grown on silicon chips can be engineered to show reflectance peaks at different visible wavelengths. Among the most significant applications of PSi in these areas are light emitting diodes (LEDs) (Canham et al., 1996), solar cells (Menna et al., 1995), Bragg reflectors (Pavesi and Dubos, 1997), optical waveguides (Loni et al., 1996), photodetectors (Lee et al., 1997), photonic crystals, and optical microcavities (Pellegrini et al., 1995).

## Fabrication of Porous Silicon

**Figure 3:**
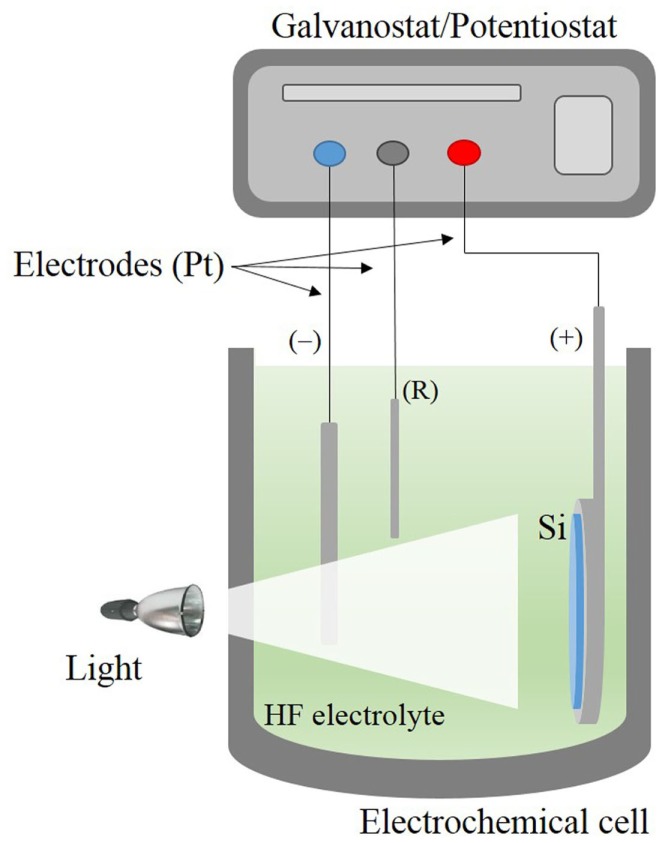
**Scheme of the electrochemical cell used for PSi formation**.

Porous silicon is nowadays almost exclusively fabricated by the electrochemical etching of silicon wafers in hydrofluoric acid-based electrolytes (Bisi et al., 2000). In this technique, the silicon wafer acts as the anode and platinum electrodes are used as cathode and counter electrodes. The system is connected to a power supply, which regulates the current/voltage through the silicon crystal (Kolasinski, 2005). Due to the fact that hydrofluoric acid is extremely corrosive, Teflon beakers are commonly used as reactors. The electrochemical process is mainly controlled by the current/voltage and solution composition. A scheme of a typical electrochemical cell commonly utilized is shown in Figure 3.

**Figure 4:**
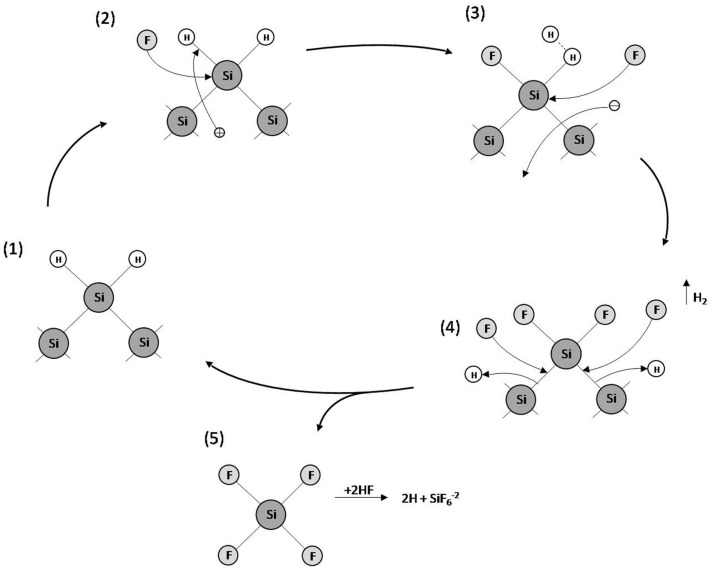
**Model of silicon dissolution/PSi formation**.

Many different models have been proposed to explain pore formation in PSi. However, the most accepted model concerning the silicon dissolution/PSi formation is the series of electrochemical reactions schematized in Figure 4 (Lehmann and Gosele, 1991). Initially, the Si atoms on the surface are passivated by Si–H bonds (1). Afterwards, holes are injected from the bulk to the Si surface by the power supply. Thus, a nucleophilic attack on Si–H bonds by F^−^ anions can occur and Si–F bonds are formed (2). The Si–F bonds cause a polarization effect allowing a second F^−^ anion to attack and replace the remaining hydrogen bonds. Two hydrogen atoms can then combine, injecting an electron into the substrate (3). The polarization induced by the Si–F bonds reduces the electron density of the remaining Si–Si backbonds making them susceptible to further attack by HF in such a manner that the remaining silicon surface atoms are bonded to the hydrogen atoms, which suffer a second nucleophilic attack by a F^−^ anion forming silicon tretrafluoride (SiF_4_) (4). The SiF_4_ molecule reacts with HF to form the highly stable ${\mathrm{SiF}}_{6}^{2-}$ fluoroanion. Finally, the surface returns to its “neutral” state until another hole is available (5).

**Figure 5:**
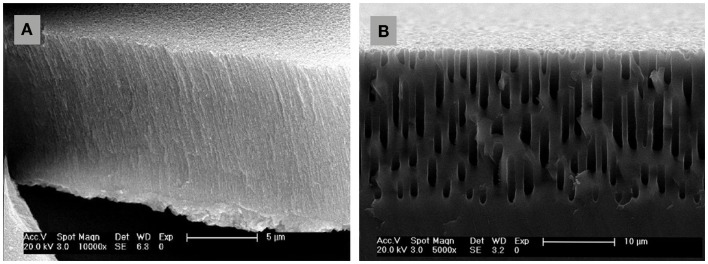
**Field emission scanning electron microscopy images of columnar PSi**. **(A)** nanostructured (thickness ~10 μm and pore diameter ~30 nm), and **(B)** microstructured (thickness ~20 μm and pore diameter ~1 μm).

All the key properties of PSi/nanoPS including porosity, thickness, pore diameter, and microstructure, depend on the properties of the Si wafer and the synthesis parameters (Bisi et al., 2000). These parameters include HF concentration, current density, wafer type and resistivity, reaction time, illumination (mainly in the case of n-type Si wafers), temperature, and drying/post-formation process. In Table 1, the main effects of the various synthesis parameters on the properties of the resulting PSi/nanoPS layers are summarized. Accordingly, a wide variety of structures are obtained depending on the fabrication parameters (Smith and Collins, 1992), including nanoPS. As an example of this, Figure 5 shows two different columnar PSi layers with feature sizes in the range of nanometers and microns.

**Table 1:** **Main effects of the synthesis parameters on PSi formation**.

An increase of … yields a	Porosity	Etching rate	Critical current
HF concentration	Decreasing	Decreasing	Decreasing
Current density	Increasing	Increasing	–
Anodization time	Increasing	Almost constant	–
Temperature	–	–	Increasing
Wafer doping (p-type)	Decreasing	Increasing	Increasing
Wafer doping (n-type)	Increasing	Increasing	–

*Reprinted from Bisi et al. (2000)*.

## Nanostructured Porous Silicon in Life Sciences: Biomarkers

**Figure 6:**
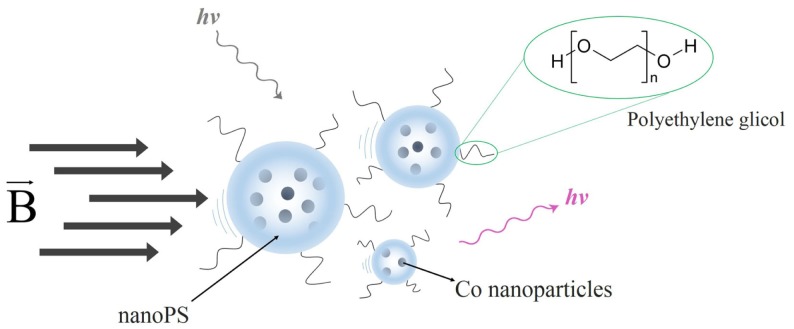
**Schematic representation of nanoPS-based hybrid luminescent/magnetic nanostructured particles (hlmNPs) after conjugation**. These comprise a nanoPS shell (striped particles) with a multicore of Co nanoparticles (solid blue particles). The hlmNPs have subsequently been conjugated with poly(ethylene glycol).

The typical dimensions and overall characteristics of nanoPS may lead to its use in the field of tumor imaging, given the great potential hold by nanomaterials that can circulate in the body to diagnose disease. Additionally, the growth of magnetic nanoparticles into the porous structure would lead to hybrid systems thus add extra functionalities to them. As a single example in this line, hybrid particles were fabricated by the growth of Co nanoparticles into nanoPS (Figure 6), leading to both luminescent and magnetic properties, i.e., intense luminescence combined with magnetic response (Munoz-Noval et al., 2011). The resulting hybrid particles were subsequently conjugated with polyethylene glycol (PEG), aiming at increasing the hydrophilic properties of the particles and opening the way to PEGylation mechanisms for the formation of targetable biomolecular-particle complexes. MTT cytotoxicity assays in hMSC cultures proved the low toxicity of the hybrid particles. The possibility to fabricate silicon-based particles with dual magnetic/luminescent properties opens a wide range of applications in the field of biomedicine. On the one hand, the versatility of the particles can be increased by varying the size and/or composition of nanoPS to obtain customizable luminescence (i.e., variable color) and magnetic behavior. On the other hand, given the versatility of silicon chemistry, several functional groups can be attached to the nanoPS-based particles and various biomolecules immobilized in order to provide internal specificity within the cell (selective organelle labeling) or even applications in combined deep-tissue imaging.

The overall magnetic behavior of the nanoPS-based hybrid nanoparticles can be tuned by changing the porosity, type, and/or size of the pores of nanoPS, given that those parameters determine the size and distribution of the magnetic nanoparticles inside the pores, in addition to the dipolar interactions between magnetic nanoparticles.

## Cell Scaffolds Based on Porous Silicon

As stated above, Canham (1995) demonstrated in 1995 for first time the bioactivity of PSi/nanoPS by means of the hydroxycarbonated-apatite *in vitro* growth on porous surfaces over periods of days to weeks. Since then, different bioapplications have been developed, including biosensing (Dhanekar and Jain, 2013), drug delivery (Anglin et al., 2008), tissue engineering (Coffer et al., 2005), tumor imaging (Martin-Palma et al., 2010), and bioreactor platform (Stewart and Buriak, 2000), among others. These applications mainly rely on the intrinsic large surface area and surface chemistry providing high reactivity. Hence, it is possible to generate a specific chemical composition or molecular adsorption on the surface of PSi/nanoPS (Stewart and Buriak, 2000). In addition to this, PSi is also an excellent biomaterial given its biocompatibility, biodegradability, and bioresorbability (Hernández-Montelongo et al., 2012). The appropriate bioproperties of PSi/nanoPS are largely generated due to its particular susceptibility to oxidation (Eq. 1), given that silicon oxide is readily dissolved by body fluids (Anglin et al., 2008) and later non-toxically eliminated as silicic acid in the urine (Reffitt et al., 1999) (Eq. 2).

(1) $$\text{Si}+{\text{O}}_{\text{2}}\to \text{Si}{\text{O}}_{\text{2}}$$

(2) $$\text{Si}{\text{O}}_{\text{2}}+\text{2O}{\text{H}}^{-}\to {\left[\text{Si}{\text{O}}_{\text{2}}{\left(\text{OH}\right)}_{\text{2}}\right]}^{\text{2}-}$$

Moreover, PSi/nanoPS has been also combined with other materials, introduced into its pores or deposited on its surface, leading to the development of composites (Hérino, 2000), which can improve its properties thus extending the range of applications (Anglin et al., 2008; Fernandez et al., 2009).

Cells respond to topographic features (Torres-Costa et al., 2012) and surface chemistry of substrates (Low et al., 2006) in a wide variety of ways, with a clear dependency on many factors including cell type, feature size, and geometry, and the physicochemical properties of the substrate material. As PSi/nanoPS is easily fabricated and modified by different processes, a range of biomaterials can be designed through changes in its topography and surface chemistry. It becomes an excellent substrate to support and control cell adhesion, morphology, proliferation, migration, and differentiation in different cell lines (Sun et al., 2007a). That is why the most relevant recent uses of PSi as a biomaterial are cell scaffolds. The development of PSi-based devices oriented to this application is increasingly relevant during last decade. The state of the art of it is outlined below.

Table 2 highlights different cases of cells cultured on PSi-based cell scaffolds. As in other PSi applications, the use of PSi as a cell scaffold can take place in the form of a single material, after appropriate surface modification, or as a composite biomaterial.

**Table 2:** **Cell culture on different kinds of PSi scaffolds**.

PSi scaffold	Cell culture	Cellular key results	Reference
Nanostructured and exposed to SBF	B50 neuron and Chinese hamster ovary (CHO)	CHO were adhered on high- and low-porosity PSi, no cells were found on crystalline Si. B50 cells preferred the PSi surface than poly- and bulk-silicon	Bayliss et al. (1997, 1999)
Nanostructured	B50 neuron and Chinese hamster ovary (CHO)	PSi offered significant advantages over bulk Si surfaces for cell adherence and viability	Bayliss et al. (2000)
Oxidized by ozone	Primary rat hepatocyte	Cells were available to attach, spread, and function on PSi.	Chin et al. (2001)
Thermally oxidized, carbon layer coated, hexametyldisilazaned, and Si–C deposited by hexametildisilane	Human retinal endothelial cells, mouse aortic endothelial cells, murine melanomas, neuronal mouse cells (B50), hamster ovarian cells (CHO)	All PSi substrates were appropriated for cultivating adherent cells *in vivo* and without noticeable toxicity	Angelescu et al. (2003)
Composited with polycaprolactone and exposed to SBF	Human kidney fibroplast cells	Scaffolds were non-toxic to cells and sustained the *in vitro* stability and proliferation of fibroblasts	Coffer et al. (2005), Whitehead et al. (2008), and Fan et al. (2011)
Functionalized with *N*-(triethoxysilylpropyl)-*O*-poly(ethylene oxide) urethane and micro-patterned by direct laser writing	Neuroblastoma cells	Cells growth closely mimicked the laser written micropatterns	Khung et al. (2006)
Patterned by stain etching	Rat hippocampal neuron (B50)	Cells preference adhered to PSi patterns than crystalline and polycrystalline Si. PSi surface topology influenced on proliferation of the neuron network	Sapelkin et al. (2006)
Modified by ozone oxidation, amino and polyethylene silanizated, and coated with collagen	Rat pheochromocytoma (PC12) and human lens epithelial	Scaffolds with collagen coating and amino salinization promoted cell attachment for both cell lines. Cells attached poorly to ozone oxidized and polyethylene glycol salinized PSi surfaces	Low et al. (2006)
Nano-, meso-, and macro-structured	Osteoblast cells	MacroPSi performed better than mesoPSi and nanoPSi in supporting osteoblast growth and sustaining their function	Sun et al. (2007a,b)
Structured with pore size continuous gradient	Neuroblastomas	Cells displayed morphological characteristics, which were influenced by the pore size of PSi. Cells were sensitive to nanoscale surface topography with feature sizes of 20 nm	Khung et al. (2008)
Thermally oxidized and aminosilanized	Human lens epithelial cells	Both PSi scaffolds supported the attachment and growth of human ocular cells, which were able to survive and migrate into ocular tissue spaces *in vivo*	Low et al. (2009)
Oxidized by air, H_2_O, and medium containing 10% fetal calf serum	Osteoblast cells	PSi surface reduced cell adhesion, but suitable modification using fetal calf serum increased cell adhesion	Yangyang et al. (2009)
Microparticles thermally oxidized and non-treated	Human lens epithelial cells	Non-treated PSi produced reactive oxygen species, which interacted with the components of the cell culture medium, leading to the formation of cytotoxic species. Oxidation of PSi not only mitigated, but also abolished the toxic effects	Low et al. (2010)
Encapsulated in microfibers of polycaprolactone	Human lens epithelial cells	The composite was a flexible and controlled degradable scaffold, which actively supported cells attachment. Samples beneath the conjunctiva of rat eyes without visible infection and erosion of the ocular surface	Kashanian et al. (2010)
Chemically micro-patterned by photolithography and surface silanization	Mammalian neuronal cell line	98% Total of cell attachment was on the patterned regions	Sweetman et al. (2011)
Surface modified with peptides gradients	Rat mesenchymal stem cells	Cells attachment on PSi surface increased with increasing peptides density	Clements et al. (2011)
Chemically modified by cathodic bias and coated with nano-hydroxyapatite colloid suspension	Murine macrophage cells	Modified PSi surfaces were shown to be better than unmodified PSi to be used as a support for cell culture	Sánchez et al. (2011)
Dry-etched using XeF_2_	Bone marrow-derived mesenchymal stromal cells	PSi scaffold obtained by this novel technique was available to support the replication of cells for up to 21 days in culture	Hajj-Hassan et al. (2011)
Mesoporous structured (5 and 20 nm pore size) and thermal oxidized	Primary human endothelial, mouse mesenchymal normal, mouse neuroblastoma, and human cortical neuron cell line	Surface density of the adhering cells was larger on 5 nm pore size PSi than on 20 nm pore size PSi substrates, depending on the cell type	Gentile et al. (2012)
1D nanostructured PSi micropatterns	Human mesenchymal stem cells (hMSCs)	hMSCs cultured on designed PSi-stripes exhibited a clear polarization with respect to patterns	Muñoz et al. (2012) and Punzón-Quijorna et al. (2012)
1D and 2D nanostructured PSi micropatterns	Human mesenchymal stem cells (hMSCs)	hMSCs were sensitive to 1D and 2D PSi patterns and their migration could be controlled by the particular surface topography and chemistry of scaffolds	Torres-Costa et al. (2012) and Peláez et al. (2013)
Hexagonal geometric micro-patterned	Human mesenchymal stem cells (hMSCs)	hMSCs adapted their morphology and cytoskeleton proteins from cell–cell dominant interactions at the center of the hexagonal patterns	Ynsa et al. (2014)
Composited with calcium phosphates (CaP) deposited by cyclic spin coating and cyclic electrochemical activation	Human mesenchymal stem cells (hMSCs)	The morphology appearance, active mitosis, and density of adhered cells depended on the morphology and CaP phase of composite obtained by each synthesis technique	Hernandez-Montelongo et al. (2014)
Nano-, meso-, and macro-structured modified by thermal oxidation, silanization with aminopropyltriethoxysilane (APTES), and hydrosilylation with undecenoic acid or semicarbazide	Dental pulp stem cells (DPSC)	PSi with 36 nm pore size showed the best adhesion and the fastest growth rate for DPSC compared to PSi comporting smaller pore size (10 nm) or larger pore size (1 μm), especially after silanization with APTES	Sun et al. (2007a) and Collart-Dutilleul et al. (2014)
Composited with polycaprolactone and PSi microparticles, exposed to SBF	Osteoblast cells	The addition of increasing quantities of PSi to the composite resulted in proportional increases in cell proliferation	Henstock et al. (2014)

### Surface topography and pore size influence

Different approaches have been taken to determine the nature of the interaction between PSi and living cells. Preliminary studies were mainly oriented to studying the influence of Si-based substrates in cell adhesion and viability. In fact, Si was first proposed as a very promising candidate to develop semiconductor-based biodevices given the possibility to integrate it in current Si-based technology. Subsequently, it became very popular as a non-toxic material among other semiconductors such as GaAs or InAs. The advantages of incorporating PSi/nanoPS in this research were preliminarily explored by several groups like Bayliss’s group (Bayliss et al., 2000), which evaluated the cell adhesion and viability by morphology and MTT tests in Chinese hamster ovary (CHO) cells and rat neuronal B50 cells directly cultured onto the substrate surface. Their findings pointed out to the optimal viability of B50 in untreated, pre-oxidized PSi over other Si substrates (bulk crystalline Si and nanocrystalline Si), meanwhile for the CHO cells, the polycrystalline Si showed to be the most suitable. These results highlighted the importance of surface nano-structuring in the adhesion and viability for each particular kind of cell.

Coetaneous research from the group of Prof. Bhatia explored the long-term viability of hepatic cell cultures onto nanoPS (Chin et al., 2001). They cultured primary rat hepatocytes on several substrates including tissue culture polystyrene, crystalline Si, and pre-oxidized nanoPS pre-treated with collagen I solutions to evaluate the adhesion, long-term viability, and function of the cells. Their findings pointed out to a similar viability and functionality of the cells cultured for weeks in the nanoPS substrates compared with the control polystyrene plate.

Beyond the differences between the various surface chemical compositions, surface topography plays a key role in cell-surface interaction. In this sense, the influence of PSi surface by comparing different porosities with analog chemistries on the adhesion, growth, and viability of cultured cells has been explored by several groups. One of the pioneering works in enclosing the effect of pore size in these biological parameters was carried out by culturing osteoblast and osteosarcoma cells onto PSi of scaled porosities to check the osteoconductivity (Sun et al., 2007b). This work pointed out the optimal pore scale to culture viable cells with their osteogenic functions conserved. They carried out this evaluation by a combined study of ATP metabolism, cell adhesion, and a gene expression to check the bone formation of cells cultured on PSi with three pore sizes and similar chemical composition. Gene expression experiments focused in the detection of three biomarkers for bone formation: alkaline phosphatase, osteocalcin, and type I collagen. The results for a long-term study concluded that the gene biomarkers for cells grown over macropores (ca. 1 μm) were conserved in the same level than the control, meanwhile for nanoPS (<15 nm) and mesoPS (>15 nm), the gene expression levels where kept in a moderate expression level.

**Figure 7:**
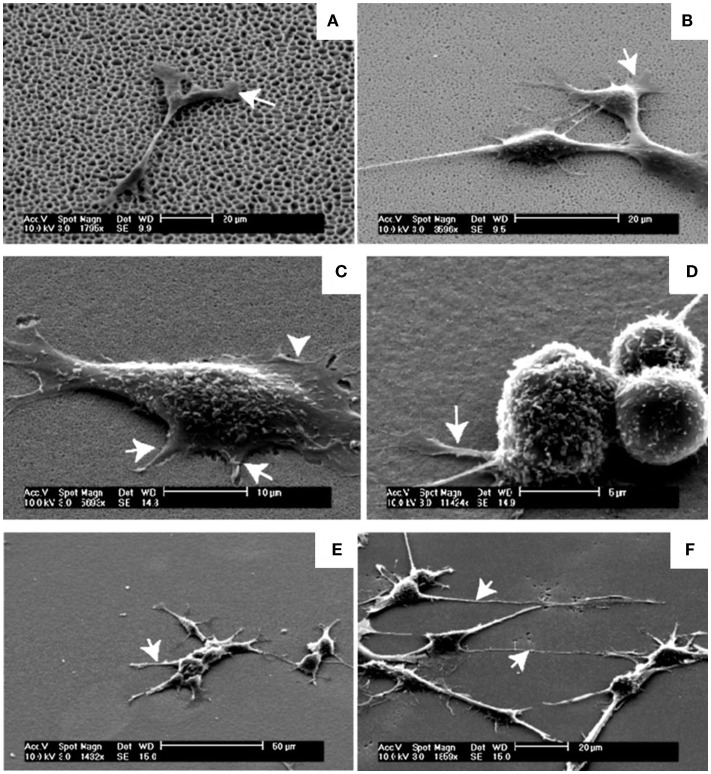
**Neuroblastoma cells growth on the graded PSi observed in SEM after 24 h-incubation time**. **(A)** SEM of neuroblastoma cells on the 1000–3000 nm region, arrow depicting the flattening of the lamellipodia serving as anchorage points. **(B)** The 300–1000 nm region, arrows highlighting the flattening of the lamellipodia closer to the main cell body. **(C)** The 100–300 nm region, shorter filopodia-like protrusions between 2 and 4 μm as indicated by the arrows while the arrowhead shows the lamellipodia. **(D)** Spherical neuroblastoma cells adhering on the 50–100 nm pore size region with relatively short and thick filopodia at the base. **(E)** The 20–50 nm region, initial recovery of the formation of neuritic processes as indicated by the arrow. **(F)** The 5–20 nm region, processes were long, spanning up to 40 μm from the cell body with significant outgrowth of filopodia as denoted by the arrows. Reprinted from Khung et al. (2008).

Another interesting work (Figure 7) was developed by Khung et al. (2008). They grew neuroblastoma cells on oxidized PSi/nanoPS with continuous gradient pore sizes ranging from the nano- to the microscale. They observed a high influence of changes in surface topography on the density and morphology of adherent cells. On PSi with pore diameter between 1000 and 3000 nm, cells were unable to adhere optimally on surfaces because pore sizes were too large for filopodia to find anchorage points on the surface. However, cells could stabilize themselves through cell–cell interactions, reducing the need for cell–PSi contact. In the case of the pore range from 1000 to 100 nm, the authors reported an increasing incidence of thin protrusions from the cell body and a shorter time for the cell–cell interaction process. On the 50–100 nm PSi region, cells had spherical morphology and regrouped forming clusters. Nevertheless, cells recover their typical neuroblastoma morphology when PSi pores were <20 nm.

As a general conclusion from these studies, the porosity and pore size as surface topographic features must be taken into account. Moreover, each problem should be boarded specifically for each type of cell, depending on the nature of the mechanical interactions of each type of cell in its parental environment.

### Chemically-modified

**Figure 8:**
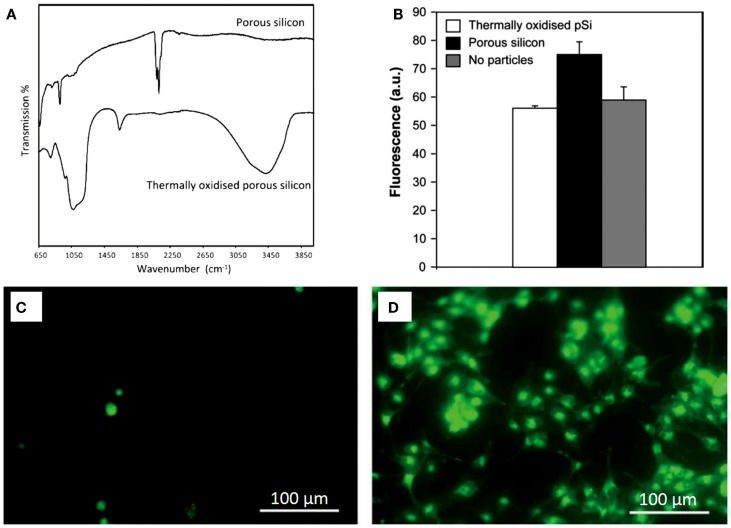
**(A)** Transmission FTIR spectra of PSi and thermally oxidized PSi. **(B)** Detection of residual oxygen species with PSi particles in 24 h-incubation of human lens epithelial cells on a non-treated PSi, and on a thermally oxidized PSi. Results are expressed as mean fluorescence units at 100 s integration time point. Fluorescence intensity is significantly higher for PSi particles in comparison to thermally oxidized PSi particles and to the no particles samples. **(C)** Twenty-four hours incubation of human lens epithelial cells on a non-treated porous silicon membrane, and **(D)** on a thermally oxidized porous silicon membrane. Reprinted from Low et al. (2010).

The nanoscale architecture of PSi is inherently fragile (Buriak et al., 1999) and shows a great reactivity due to the chemical instability of the surface just after formation. When PSi is prepared, its surface is predominantly SiH_x_-terminated and is highly reactive (Naveas et al., 2012), being this the reason why rapid modification of the surface occurs if it is not passivated (Demontis, 2006). Different chemical reactions have been used to enhance the mechanical and chemical properties of PSi (Anglin et al., 2008): oxidation, hydrosilylation, silanization (namely, amino-silation), and others. These chemical treatments modify the physicochemical properties of PSi/nanoPS improving its properties. Using human lens epithelial cells as a model, Low et al. (2010) tested the biocompatibility of non-treated and thermally oxidized PSi particles (at 600°C for 1 h). They reported a poor cell adhesion to non-treated PSi, in contrast to thermally oxidized PSi microparticles. Using the fluorescent probe 20,70-dichlorofluorescin as an indirect cell culture assay, the authors showed that non-treated PSi microparticles produced reactive oxygen species, which interacted with the components of the cell culture medium, leading to the formation of cytotoxic species. Oxidation of PSi samples not only mitigated but also abolished the toxic effects (Figure 8).

As the biocompatibility of materials is also strongly linked to their normal electrochemical potential and surface energy with respect to the living body, another technique to improve the biocompatibility of PSi is the deposition of materials with electrochemical potential and surface energy corresponding to values for living tissues. Only carbon, gold, and platinum have electrochemical potentials close to living tissue: +0.333, +0.332, and +0.334 mV, respectively. Besides, the surface energy of these elements is in the range of 20–30 mN/m, which likewise corresponds to the values for living tissue (Angelescu et al., 2003). In that sense, Angelescu et al. (2003) used different techniques to form silicon–carbon bonds on PSi: thermal treatment, carbon layer deposition, and a-SiC deposition from hexamethyldisilane. On all these PSi-based cell scaffolds, five kinds of cells were used to test their biocompatibility: human retinal endothelial cells (HREC), mouse aortic endothelial cells (MAEC), murine melanoma cells (B16-F1), neuronal mouse cells (B50), and hamster ovarian cells (CHO) were grown and studied by laser scanning cytometry (LSC). The authors reported that PSi covered with different carbon layers was appropriated for culturing adherent cells *in vivo* without noticeable toxicity. Polylysine or collagen coatings were not required to bioactivate the substrates.

**Figure 9:**
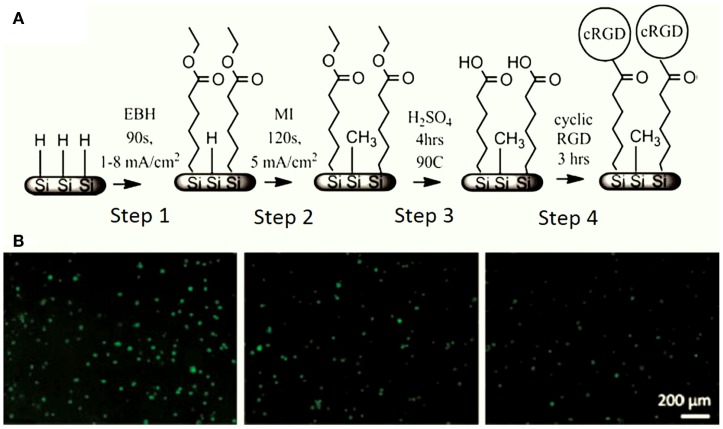
**(A)** PSi chemical modification by a four-step functionalization cascade. Step 1: electrografting of EBH, Step 2: backfilling with MI, Step 3: ester cleavage in boiling H_2_SO_4_, and Step 4: cRGD immobilization. **(B)** Rat MSC response to cRGD gradient on PSi, with the cRGD density decreasing from left to right. Reprinted from Clements et al. (2011).

Others interesting methods to improve the biocompatibility of PSi are the chemical functionalization cascades. A notable work was developed by Clements et al. (2011) using this kind of techniques. The authors generated peptide gradients on PSi via electrografting following a four steps process (Figure 9A). The PSi surface was initially functionalized with ethyl-6-bromohexanoate and backfilled with methyl iodide to generate a stable surface (Steps 1 and 2). The peptide density gradient was generated by hydrolysis of the ester groups on the surface followed by carbodiimide coupling of cRGD (cyclo Arg-Gly-Asp-d-Phe-Lys), an oligopeptide sequence found in cell adhesion structures (Steps 3 and 4). Using rat mesenchymal stem cells (MSC) for cell cultures on these modified PSi surfaces, the authors showed that cell attachment increased with increasing cRGD density electrografting (Figure 9B).

### Hydroxyapatite functionalization

In order to use PSi/nanoPS or any composite based on PSi/nanoPS as bone engineering scaffold, deposition of calcium phosphate (CaP) ceramics in its hydroxyapatite [HAP, Ca_10_(PO_4_)_6_(OH)_2_] phase has been suggested. This is primarily because HAP is the main inorganic extracellular matrix component of skeleton cells (Dorozhkin and Epple, 2002; Seong et al., 2010), supporting mobility (Dorozhkin and Epple, 2002), calcium reserve (Bertazzo et al., 2010), and its role in the regulation of metabolic energy (Wolf, 2008; Bertazzo et al., 2010; Swetha et al., 2010). HAP is a biocompatible and bioactive material, capable of guiding bone formation and providing direct chemical bonds with natural bone (Bertazzo et al., 2010; Zhu et al., 2010). After HAP implantation in the body, solubilization and ionic exchange from the HAP surface start. The solubilization of the HAP surface continues a certain period depending on the electrolytic properties of the implantation medium. When the equilibrium between physiological ions and the modified surface of HAP is reached, the adsorption of proteins and organic material can proceed. The proteinaceous biofilm formed triggers cell adhesion and proliferation and can initiate the production of new bone. That is why different works were focused on the synthesis of PSi/HAP cell scaffolds.

The most common processes for PSi/nanoPS functionalization of CaP are the biomimetic growth using simulated body fluids (SBF), and electrodeposition techniques using CaP aqueous solutions (Hernández-Montelongo et al., 2012). When PSi/nanoPS is exposed to SBF or CaP solutions, it forms silicic acids, which then polycondensate to form particles of silica and apatite (Henstock et al., 2014). Besides, favored sites of CaP nucleation are given by the surface roughness of PSi (Hernández-Montelongo et al., 2012).

**Figure 10:**
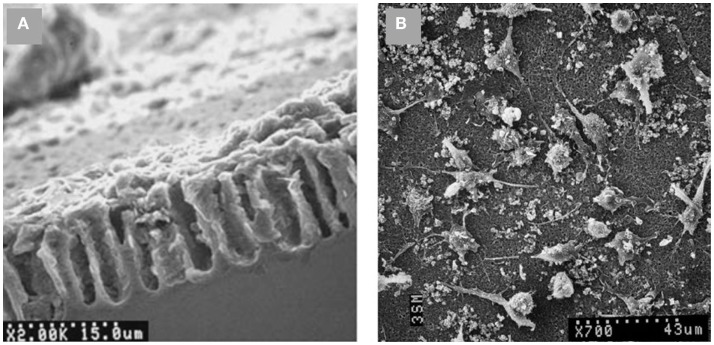
**SEM images of (A) PSi–HAP scaffold (cross-sectional view), and (B) murine macrophages on PSi–HAP scaffold**. Reprinted from Sánchez et al. (2011).

Different cell assays have been carried out on PSi-based scaffolds after SBF exposition. As an example, Bayliss et al. (1997, 1999) successfully grew B50 neurons and Chinese hamster ovary cells, being one the firsts reported *in vitro* experiments. Using an electrodeposition technique, Sánchez et al. (2011) synthetized a PSi-HAP scaffold, which successfully supported murine macrophage cells (Figure 10). More recently, Hernandez-Montelongo et al. (2014), using also an electrochemical deposition and a sol–gel technique, obtained two different kinds of PSi–CaP scaffolds. The authors showed that the morphology, active mitosis, and density of adhered bone-derived progenitor cells depended on the morphology and CaP phase obtained by each technique.

### PSi-polycaprolactone composites

**Figure 11:**
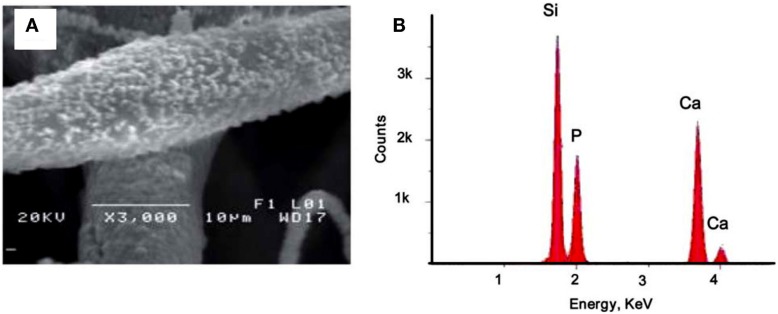
**(A)** 5% PSi–PCL composite fibers soaked in SBF for 3 weeks. The fiber surface was deposited with calcium phosphate nanocrystals upon SBF exposure. **(B)** EDX analysis confirms the composition of the nanoparticles are calcium and phosphorous. Reprinted from Fan et al. (2011).

Another strategy to support the proliferation, viability, adhesion, and differentiation of bone precursor cells on PSi-based scaffolds is to form composites with polycaprolactone (PCL) (Coffer et al., 2005; Whitehead et al., 2008; Henstock et al., 2014). PCL is a non-toxic biodegradable polyester, which has a longer degradation time (>6 months) than other biodegradable polymers commonly used in tissue engineering applications (Whitehead et al., 2008). PCL also allows cell growth and infiltration by newly forming bone cells, such as osteoblasts. Different groups have recently synthesized cell scaffolds by compositing PSi with PCL. Coffer et al. (2005) and Whitehead et al. (2008) could successfully sustain the *in vitro* stability and proliferation of fibroblasts for 7 days on PSi–PCL scaffolds after SBF exposition. Fan et al. (2011), in a similar work, extended the cell culture time to 14 days and their results were also satisfactory. Figure 11A shows typical morphologies of calcium phosphate nodules formed on PCL fibers containing 5% encapsulated PSi as produced by Fan et al. (2011). Such nucleation is induced after a SBF exposure period of 3 weeks. The associated EDX spectrum in Figure 11B confirmed the presence of calcium and phosphorous. The calcium/phosphorous ratio of samples was in the range from 1.5 to 1.7, which suggested a mixture of phases including octacalcium phosphate and hydroxyapatite.

**Figure 12:**
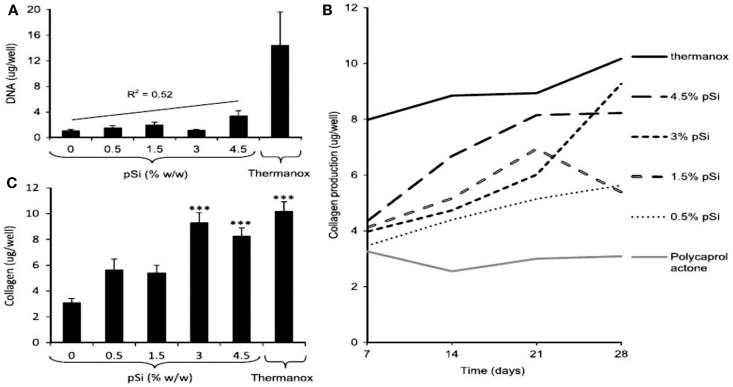
**The activity of human osteoblasts on the surface of PCL, PSi–PCL composites, and thermanox coverslips (a tissue culture plastic) was compared**. **(A)** After 28 days, the DNA content of lysates from cells cultured on composites was lower than Thermanox controls, but proportional to the amount of PSi in the composite (gradient: 182 ng DNA/mg PSi). **(B)** The production of collagen by osteoblasts was again highest on Thermanox, and lowest on the PCL-only disk; increasing PSi content in the composite resulted in an increased rate of collagen production. **(C)** After 28 days, the amount of collagen produced on composites containing 3 or 4.5% pSi was significantly higher than PCL-only and equivalent to the amount produced on Thermanox. *Error bars* show SEM, *n* = 6 (composites), *n* = 12 (Thermanox) (***P = 0.001). Reprinted from Henstock et al. (2014).

More recently, Henstock et al. (2014) made composites of PSi with PCL to enhance the deposition of hydroxyapatite on the surface of the composite (Figure 12). This enhancement was found to be proportional to the increase of PSi content. Furthermore, silicon-substituted hydroxyapatites appear to spontaneously form on the composites in simulated body fluid. The osteoblast proliferation was measured by DNA quantification. Proliferation on the composite materials was directly proportional to PSi content, with 0.5% PSi composites supporting 40% more osteoblasts than the blank controls and 4.5% PSi composites supporting 212% more cells than PCL alone. Besides, the collagen production by the osteoblasts was lower on pure PCL and increased proportionally with PSi content in the composite (Figures 12B,C).

### Micro-patterned porous silicon

The electrochemical technique used for the fabrication of PSi allows the generation of lateral and vertical patterns of porosity, thus increasing the versatility of this material. Patterned and textured surfaces at the micron- and nano-scales with very different chemical and topographic characteristics can be fabricated and used to control cell–substrate interactions and regulate/condition cell function. The use of a diversity of lithographic methods has allowed the fabrication of sets of PSi/nanoPS micropatterns bearing contrasting properties toward cell adhesion and thus inducing polarization, migration, and differentiation.

**Figure 13:**
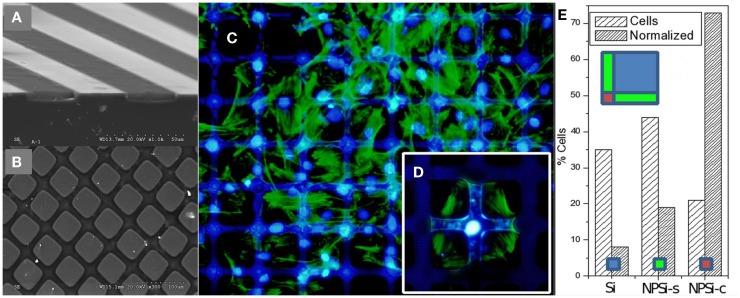
**Perspective SEM images from a cross section performed in micropatterns showing: (A) alternating Si and PSi stripes, and (B) Si/PSi square grids**. **(C)** Fluorescence microscopy images of human mesenchymal stem cells (hMSCs) on 100 μm Si/25 μm nanoPS square micropatterns. Actin is stained green and nuclei are stained blue. **(D)** Detailed image at an intersection, and **(E)** histogram of hMSC population from image **(C)** with absolute % and area normalized population (left and right columns, respectively). “Si” refers to silicon areas, “NPSi-s” to PSi stripes, and “NPSi-c” to PSi “crossways.” Reprinted from Torres-Costa et al. (2012).

As an example, Figure 13 shows 1D and 2D patterns with well-defined nanoPS regions grown on the surface of Si wafers, which were fabricated by ion beam irradiation and subsequent electrochemical etch. These chemically and morphologically patterned surfaces have been exploited to control the surface distribution and shape of human skeletal progenitor cells and, at the same time, to study cell adhesion and migration characteristics (Torres-Costa et al., 2012). The experimental results (Figure 13) show that human mesenchymal stem cells (hMSCs) are sensitive to surface patterns and that migration can be controlled, so that cells arrange in response to the particular surface topography and chemistry. In particular, it has been observed that aging of nanoPS-based micropatterns in physiological conditions gives rise to a surface finishing with contrasting properties (Muñoz et al., 2012). NanoPS presents a highly oxidized and hydroxylated nanostructured surface showing extremely hydrophilic behavior. The surface chemical contrasts are sensed by hMSCs, which tend to orientate according to Si/nanoPS stripes. Their adhesion is inhibited on nanoPS so that they assemble preferentially on Si areas. However, in the case of 1D patterns, the reduction of the Si stripe width favors the adhesion of the actin cytoskeleton on two parallel Si stripes with the nucleus standing on nanoPS.

In an alternative approach, single-pulse UV laser interference has also been used to fabricate 1D and 2D diffractive patterns in nanoPS with different shapes and a wide range of periodicities in relatively large areas (up to a few square millimeters) (Peláez et al., 2013). The patterns are formed by alternate regions of almost unaltered PSi and areas where PSi has melted and converted into Si nanoparticles. It was observed that the hMSCs bind directly and align along the transformed regions of the pattern whenever the width of the trenches on these regions compares with the dimensions of the hMSCs. The morphology of the adhered hMSCs is consistent with their active polarization.

Meanwhile, the previous works show that the induced nanoPS micropatterns allow controlling polarization and migration, the presence of asymmetric periodic nanoPS motives was proposed to control the sense of migration. Columns of equilateral Si triangles in a PSi background were fabricated. hMSCs in a central Si seeding area were observed to select pointing out arrows to initiate a radial escape migration. Relevantly, markers for bone differentiation (Runx2 and vitamin D receptor) were morphologically different for hMSCs in the central seeding area and those migrating on the Si/PSi triangle columns (Punzón-Quijorna et al., 2012). This is a clear example of how engineered surface features can induce phenotype alterations by a morphological reprograming.

## PSi Photo-Optical Properties Applied in Cell Scaffolds

**Figure 14:**
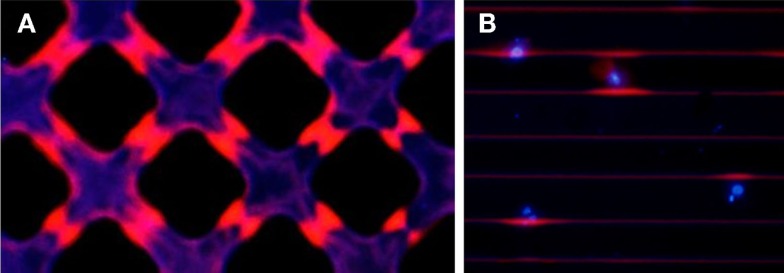
**Fluorescence microscopy image (red + blue channels) of (A) a crossing stripe micro-patterned PSi exposed to phosphate buffered saline for 4 h to show the differentiated aging caused by the porosity gradients, and (B) hMSCs cultured for 24 h on a PSi stripe micropattern showing colocalized red intensity with the presence of the cells (identified by nuclear DAPI staining)**.

Porous silicon topographic micropatterns for tissue engineering are ideal structures for the identification of photoluminescence evolution in PSi upon physiological exposure. The formation of the micropatterns creates lateral resistivity gradients in Si, which translate, upon PSi formation, into lateral porosity gradients at the Si/PSi interface. When immersed in physiological fluids, such gradients induce differential aging rates of PSi, and thus observation of the surface with a fluorescence microscope at particular timescales shows contrasting luminescence spectra, both in intensity and wavelength. Figure 14A shows a blue and red channel image of PSi crossing stripe micropatterns exposed to phosphate buffered saline during 4 h. It is patent that the center of the channels (more prone to aging) has lost the typical red fluorescence of fresh PSi related to the presence of quantum sized Si crystals. On the other hand, the areas closer to bulk Si still keep such red luminescence.

Such differentiated aging has been shown to be biologically functional in hMSC cultures. It is herein shown that the presence of cells on PSi micropatterns plays a relevant protection against aging. Figure 14B presents a blue and red channel fluorescence image of hMSCs cultured on PSi for 24 h. hMSCs were stained with DAPI to identify the nuclei of cells and localize them on the platforms. Such blue intensity in Figure 14B perfectly correlates with the presence of PSi/Si edges conserving an enhanced red luminesce, which appears only succinctly in the edges not occupied by cultured cells. It can be deduced thus that the cellular membrane establishes a contact with the surface of PSi and acts as a protection barrier against the oxidation effect of the salt ions present in the culture medium. From the technological point of view, this opens the possibility of utilization of Si/PSi micropatterns as non-labeled cell tracers during the first hours of cell culture.

## Concluding Remarks and Future Perspectives

Porous silicon is an excellent biomaterial given its biocompatibility, biodegradability, and bioresorbability, which has led to its use in different bioapplications, namely biosensing, drug delivery, tissue engineering, tumor imaging, bioreactor platform, among others. In the specific field of tissue engineering, PSi/nanoPS has been mainly incorporated as cell scaffold. Its micro/nano-morphology can regulate cell behavior and its flexible surface chemistry can be tailored to improve the PSi-cell interaction. Although first *in vitro* studies regarding the behavior of cells on PSi surfaces started almost 20 years ago, most of half of the works were reported in the last 5 years. This tendency could be explained not only for the interesting biomedical properties of PSi/nanoPS but also because PSi/nanoPS is potentially marketable due to its compatibility with high-tech electronic industry.

In this work, the use of PSi/nanoPS as a cell scaffold was extensively reviewed. For that application, PSi/nanoPS can take place in the form of a single material, after appropriate surface modification, or as a composite biomaterial. In that sense, diverse cell cultures have been studied. Although all the works showed the ability of different forms of PSi to support cell growth, which makes them good candidates for tissue engineering in general, few works were extended to *in vivo* experiments. Therefore, in order to show the biocompatibility of PSi-based cell scaffold in tissues, *in vivo* assays are mandatory since more parameters are involved.

Currently, some PSi applications have been developed to such a degree that materials, composites, and devices based on PSi are spanning to the commercial dimension. The most highlighted case is on the area of drug delivery. pSiMedica Ltd. (UK), Silicon Kinetics (USA), and pSivida (USA) are private companies dedicated to manufacture and market sustained-release drug-delivery systems based on PSi. In that sense, further research on PSi-based cell scaffolds should be performed to promote the transfer to real applied devices.

The future of Psi-based scaffolds for tissue engineering passes first by the fabrication of new PSi surface terminations. Freshly produced PSi is extremely hydrophobic, though its natural degradation in physiological environment turns it into hydrophilic in a few minutes (Hajj-Hassan et al., 2011). Paradoxically, retaining such hydrophobic behavior may be relevant for biocompatibility enhancement in certain environments, such as endothelial tissues. A stable hydrophobic PSi structure may be attainable with alkyl and fluorosilane monolayers that could, in fact, induce a completely new protein configuration upon adsorption of plasma proteins. Second, a bunch of inorganic composites with Sr and Mg rich apatite shall be studied in view of their potential osteoinductive improvement that could induce synergies with silicic acid delivery from PSi. Other relevant inorganic (non-apatitic) composites can be conceived, thinking mainly in the possible effects of sulfate ceramics in the regeneration of cartilage tissues. However, most relevant contributions may be expected from hybrid composites, especially with natural polymers, such as hyaluronan, chitosan, or cyclodextrins. This would open the possibility of envisaging synergistic drug delivery/scaffolding effects. However, the exploitation of its integrality with Si-based micro and optoelectronic devices is the most challenging. Integration with electrical/electronic circuits may help in the fabrication of signal triggered reprograming and intrinsic optical stimuli could be produced, for instance, for the activation of molecular factors intervening in cell differentiation.

## Author Contributions

JH-M drafted the initial manuscript. AM-N, JG-R, VT-C, RJM-P, and MM-S made contributions and modifications according to their field of expertise. All authors reviewed and approved the manuscript.

## Conflict of Interest Statement

The authors declare that the research was conducted in the absence of any commercial or financial relationships that could be construed as a potential conflict of interest.

## References

[B1] Angelescu A. Kleps I. Mihaela M. Simion M. Neghina T. Petrescu S. (2003). Porous silicon matrix for applications in biology. Rev. Adv. Mater. Sci. 5, 440–449.

[B2] Anglin E. J. Cheng L. Freeman W. R. Sailor M. J. (2008). Porous silicon in drug delivery devices and materials. Adv. Drug Deliv. Rev. 60, 1266–1277. 10.1016/j.addr.2008.03.017 18508154 PMC2710886

[B3] Bayliss S. Buckberry L. Harris P. Rousseau C. (1997). Nanostructured semiconductors: compatibility with biomaterials. Thin Solid Films 297, 308–310 10.1016/S0040-6090(96)09416-3

[B4] Bayliss S. Buckberry L. Harris P. Tobin M. (2000). Nature of the silicon-animal cell interface. J. Porous Mater. 7, 191–195 10.1023/A:1009686704506

[B5] Bayliss S. C. Heald R. Fletcher D. I. Buckberry L. D. (1999). The culture of mammalian cells on nanostructured silicon. Adv. Mater. 11, 318–321 10.1002/(SICI)1521-4095(199903)11:4<318::AID-ADMA318>3.0.CO;2-Z

[B6] Bertazzo S. Zambuzzi W. F. Campos D. D. P. Ogeda T. L. Ferreira C. V. Bertran C. A. (2010). Hydroxyapatite surface solubility and effect on cell adhesion. Colloids Surf. B Biointerfaces 78, 177–184. 10.1016/j.colsurfb.2010.02.027 20362420

[B7] Bisi O. Ossicini S. Pavesi L. (2000). Porous silicon: a quantum sponge structure for silicon based optoelectronics. Surf. Sci. Rep. 38, 1–126 10.1016/S0167-5729(99)00012-6

[B8] Buriak J. M. Stewart M. P. Geders T. W. Allen M. J. Choi H. C. Smith J. (1999). Lewis acid mediated hydrosilylation on porous silicon surfaces. J. Am. Chem. Soc. 121, 11491–11502 10.1021/ja992188w

[B9] Canham L. (1990). Silicon quantum wire array fabrication by electrochemical and chemical dissolution of wafers. Appl. Phys. Lett. 57, 1046–1048 10.1063/1.103561

[B10] Canham L. Cox T. Loni A. Simons A. (1996). Progress towards silicon optoelectronics using porous silicon technology. Appl. Surf. Sci. 102, 436–441 10.1016/0169-4332(96)00094-3

[B11] Canham L. T. (1995). Bioactive silicon structure fabrication through nanoetching techniques. Adv. Mater. 7, 1033–1037 10.1002/adma.19950071215

[B12] Chin V. Collins B. E. Sailor M. J. Bhatia S. N. (2001). Compatibility of primary hepatocytes with oxidized nanoporous silicon. Adv. Mater. 13, 1877 10.1002/1521-4095(200112)13:24<1877::AID-ADMA1877>3.0.CO;2-4

[B13] Clements L. R. Wang P. Harding F. Tsai W. Thissen H. Voelcker N. H. (2011). Mesenchymal stem cell attachment to peptide density gradients on porous silicon generated by electrografting. Physica Status Solidi A 208, 1440–1445 10.1002/pssa.201000320

[B14] Coffer J. L. Whitehead M. A. Nagesha D. K. Mukherjee P. Akkaraju G. Totolici M. (2005). Porous silicon-based scaffolds for tissue engineering and other biomedical applications. Physica Status Solidi A 202, 1451–1455 10.1002/pssa.200461134

[B15] Collart-Dutilleul P. Y. Secret E. Panayotov I. Deville de Périère D. Martín-Palma R. J. Torres-Costa V. (2014). Adhesion and proliferation of human mesenchymal stem cells from dental pulp on porous silicon scaffolds. ACS Appl. Mater. Interfaces 6, 1719–1728 10.1021/am4046316 24428409

[B16] Demontis V. (2006). Porous Silicon Applications in Biotechnology. Ph.D. thesis, Università degli Studi di Cagliari, Cagliari.

[B17] Dhanekar S. Jain S. (2013). Porous silicon biosensor: current status. Biosens. Bioelectron. 41, 54–64. 10.1016/j.bios.2012.09.045 23122704

[B18] Dorozhkin S. V. Epple M. (2002). Biological and medical significance of calcium phosphates. Angew. Chem. Int. Ed. 41, 3130–3146. 10.1002/1521-3773(20020902)41:17<3130::AID-ANIE3130>3.0.CO;2-1 12207375

[B19] Fan D. Akkaraju G. R. Couch E. F. Canham L. T. Coffer J. L. (2011). The role of nanostructured mesoporous silicon in discriminating in vitro calcification for electrospun composite tissue engineering scaffolds. Nanoscale 3, 354–361. 10.1039/c0nr00550a 21107480

[B20] Fauchet P. M. (1996). Photoluminescence and electroluminescence from porous silicon. J. Lumin. 70, 294–309 10.1016/0022-2313(96)82860-2

[B21] Fernandez R. E. Stolyarova S. Chadha A. Bhattacharya E. Nemirovsky Y. (2009). MEMS composite porous silicon/polysilicon cantilever sensor for enhanced triglycerides biosensing. IEEE Sens. J 9, 1660–1666 10.1109/JSEN.2009.2030643

[B22] Gentile F. La Rocca R. Marinaro G. Nicastri A. Toma A. Paonessa F. (2012). Differential cell adhesion on mesoporous silicon substrates. ACS Appl. Mater. Interfaces 4, 2903–2911. 10.1021/am300519a 22583790

[B23] Granitzer P. Rumpf K. (2010). Porous silicon – a versatile host material. Materials 3, 943–998 10.3390/ma3020943

[B24] Hajj-Hassan M. Khayyat-Kholghi M. Wang H. Chodavarapu V. Henderson J. E. (2011). Response of murine bone marrow-derived mesenchymal stromal cells to dry-etched porous silicon scaffolds. J. Biomed. Mater. Res. A. 99A, 269–274. 10.1002/jbm.a.33103 21858915

[B25] Halimaoui A. Oules C. Bomchil G. Bsiesy A. Gaspard F. Herino R. (1991). Electroluminescence in the visible range during anodic oxidation of porous silicon films. Appl. Phys. Lett. 59, 304–306 10.1063/1.105578

[B26] Henstock J. Ruktanonchai U. Canham L. Anderson S. (2014). Porous silicon confers bioactivity to polycaprolactone composites in vitro. J. Mater. Sci. Mater. Med. 25, 1087–1097. 10.1007/s10856-014-5140-5 24398914

[B27] Hérino R. (2000). Nanocomposite materials from porous silicon. Mater. Sci. Eng. B 69, 70–76 10.1016/S0921-5107(99)00269-X

[B28] Hernandez-Montelongo J. Gallach D. Naveas N. Torres-Costa V. Climent-Font A. García-Ruiz J. (2014). Calcium phosphate/porous silicon biocomposites prepared by cyclic deposition methods: spin coating vs electrochemical activation. Mater. Sci. Eng. C 34, 245–251. 10.1016/j.msec.2013.09.022 24268256

[B29] Hernández-Montelongo J. Muñoz-Noval A. Torres-Costa V. Martín-Palma R. Manso-Silvan M. (2012). Cyclic calcium phosphate electrodeposition on porous silicon. Int. J. Electrochem. Sci. 7, 1840–1851.

[B30] Kashanian S. Harding F. Irani Y. Klebe S. Marshall K. Loni A. (2010). Evaluation of mesoporous silicon/polycaprolactone composites as ophthalmic implants. Acta Biomater. 6, 3566–3572. 10.1016/j.actbio.2010.03.031 20350620

[B31] Khung Y. Barritt G. Voelcker N. (2008). Using continuous porous silicon gradients to study the influence of surface topography on the behaviour of neuroblastoma cells. Exp. Cell Res. 314, 789–800. 10.1016/j.yexcr.2007.10.015 18054914

[B32] Khung Y. Graney S. D. Voelcker N. H. (2006). Micropatterning of porous silicon films by direct laser writing. Biotechnol. Prog. 22, 1388–1393. 10.1021/bp060115s 17022678

[B33] Kolasinski K. W. (2005). Silicon nanostructures from electroless electrochemical etching. Curr. Opin. Solid State Mater. Sci. 9, 73–83. 10.1002/smll.201200175 22549930

[B34] Lee M. Wang Y. Chu C. (1997). High-sensitivity porous silicon photodetectors fabricated through rapid thermal oxidation and rapid thermal annealing. IEEE J. Quantum Electron. 33, 2199–2202 10.1109/3.644102

[B35] Lehmann V. Gosele U. (1991). Porous silicon formation: a quantum wire effect. Appl. Phys. Lett. 58, 856–858 10.1063/1.104512

[B36] Loni A. Canham L. Berger M. Arens-Fischer R. Munder H. Luth H. (1996). Porous silicon multilayer optical waveguides. Thin Solid Films 276, 143–146 10.1016/0040-6090(95)08075-9

[B37] Low S. P. Voelcker N. H. Canham L. T. Williams K. A. (2009). The biocompatibility of porous silicon in tissues of the eye. Biomaterials 30, 2873–2880. 10.1016/j.biomaterials.2009.02.008 19251317

[B38] Low S. P. Williams K. A. Canham L. T. Voelcker N. H. (2006). Evaluation of mammalian cell adhesion on surface-modified porous silicon. Biomaterials 27, 4538–4546. 10.1016/j.biomaterials.2006.04.015 16707158

[B39] Low S. P. Williams K. A. Canham L. T. Voelcker N. H. (2010). Generation of reactive oxygen species from porous silicon microparticles in cell culture medium. J. Biomed. Mater. Res. A. 93, 1124–1131. 10.1002/jbm.a.32610 19768791

[B40] Martín-Palma R. Pascual L. Herrero P. Martínez-Duart J. (2002). Direct determination of grain sizes, lattice parameters, and mismatch of porous silicon. Appl. Phys. Lett. 81, 25–27 10.1063/1.1491007

[B41] Martin-Palma R. J. Manso-Silvan M. Torres-Costa V. (2010). Biomedical applications of nanostructured porous silicon: a review. J. Nanophotonics 4, 1–20 10.1117/1.3496303

[B42] Menna P. Di Francia G. La Ferrara V. (1995). Porous silicon in solar cells: a review and a description of its application as an AR coating. Solar Energy Mater. Solar Cells 37, 13–24 10.1016/0927-0248(94)00193-6

[B43] Muñoz A. Sánchez V. Punzón E. Torres V. Gallach D. González L. (2012). Aging of porous silicon in physiological conditions: cell adhesion modes on scaled 1D micropatterns. J. Biomed. Mater. Res. A. 100, 1615–1622. 10.1002/jbm.a.34108 22447651

[B44] Munoz-Noval A. Sánchez-Vaquero V. Torres-Costa V. Gallach D. Ferro-Llanos V. Serrano J. J. (2011). Hybrid luminescent/magnetic nanostructured porous silicon particles for biomedical applications. J. Biomed. Opt. 16, 025002–025002–8. 10.1117/1.3533321 21361682

[B45] Naveas N. Costa V. T. Gallach D. Hernandez-Montelongo J. Palma R. J. M. Garcia-Ruiz J. P. (2012). Chemical stabilization of porous silicon for enhanced biofunctionalization with immunoglobulin. Sci. Technol. Adv. Mater. 13, 045009 10.1088/1468-6996/13/4/045009 PMC5090565 27877509

[B46] Parkhutik V. (2000). Analysis of publications on porous silicon: from photoluminescence to biology. J. Porous Mater. 7, 363–366 10.1023/A:1009607409050

[B47] Pavesi L. Dubos P. (1997). Random porous silicon multilayers: application to distributed Bragg reflectors and interferential Fabry-Perot filters. Semicond. Sci. Technol. 12, 570–575 10.1088/0268-1242/12/5/009

[B48] Peláez R. Afonso C. Vega F. Recio-Sánchez G. Torres-Costa V. Manso-Silván M. (2013). Laser fabrication of porous silicon-based platforms for cell culturing. J. Biomed. Mater. Res. B 101, 1463–1468. 10.1002/jbm.b.32966 24591224

[B49] Pellegrini V. Tredicucci A. Mazzoleni C. Pavesi L. (1995). Enhanced optical properties in porous silicon microcavities. Phys. Rev. B 52, R14328 10.1103/PhysRevB.52.R14328 9980751

[B50] Petrova E. Bogoslovskaya K. Balagurov L. Kochoradze G. (2000). Room temperature oxidation of porous silicon in air. Mater. Sci. Eng. B 69, 152–156. 10.1039/c3cp53618a 24158512

[B51] Punzón-Quijorna E. Sánchez-Vaquero V. Muñoz-Noval Á Pérez-Roldán M. J. Martín-Palma R. J. Rossi F. (2012). Nanostructured porous silicon micropatterns as a tool for substrate-conditioned cell research. Nanoscale Res. Lett. 7, 396. 10.1186/1556-276X-7-396 22799489 PMC3458952

[B52] Reffitt D. M. Jugdaohsingh R. Thompson R. P. Powell J. J. (1999). Silicic acid: its gastrointestinal uptake and urinary excretion in man and effects on aluminium excretion. J. Inorg. Biochem. 76, 141–147. 10.1016/S0162-0134(99)00126-9 10612067

[B53] Sánchez A. González J. García-Piñeres A. Montero M. L. (2011). Nano-hydroxyapatite colloid suspension coated on chemically modified porous silicon by cathodic bias: a suitable surface for cell culture. Physica Status Solidi C 8, 1898–1902 10.1002/pssc.201000023

[B54] Sapelkin A. V. Bayliss S. C. Unal B. Charalambou A. (2006). Interaction of B50 rat hippocampal cells with stain-etched porous silicon. Biomaterials 27, 842–846. 10.1016/j.biomaterials.2005.06.023 16098578

[B55] Seong J. M. Kim B. Park J. Kwon I. K. Mantalaris A. Hwang Y. (2010). Stem cells in bone tissue engineering. Biomed. Mater. 5, 062001. 10.1088/1748-6041/5/6/062001 20924139

[B56] Smith R. Collins S. (1992). Porous silicon formation mechanisms. J. Appl. Phys. 71, R1–R22 10.1063/1.350839

[B57] Stewart M. P. Buriak J. (2000). Chemical and biological applications of porous silicon technology. Adv. Mater. 12, 859–869 10.1002/1521-4095(200006)12:12<859::AID-ADMA859>3.0.CO;2-0

[B58] Sun W. Puzas J. E. Sheu T. Liu X. Fauchet P. M. (2007a). Nano-to microscale porous silicon as a cell interface for bone-tissue engineering. Adv. Mater. 19, 921–924 10.1002/adma.200600319

[B59] Sun W. Puzas J. E. Sheu T. Fauchet P. M. (2007b). Porous silicon as a cell interface for bone tissue engineering. Physica Status Solidi A 204, 1429–1433 10.1002/pssa.200674377

[B60] Sweetman M. J. Shearer C. J. Shapter J. G. Voelcker N. H. (2011). Dual silane surface functionalization for the selective attachment of human neuronal cells to porous silicon. Langmuir 27, 9497–9503. 10.1021/la201760w 21678982

[B61] Swetha M. Sahithi K. Moorthi A. Srinivasan N. Ramasamy K. Selvamurugan N. (2010). Biocomposites containing natural polymers and hydroxyapatite for bone tissue engineering. Int. J. Biol. Macromol. 47, 1–4. 10.1016/j.ijbiomac.2010.03.015 20361991

[B62] Torres-Costa V. Martínez-Muñoz G. Sánchez-Vaquero V. Muñoz-Noval Á González-Méndez L. Punzón-Quijorna E. (2012). Engineering of silicon surfaces at the micro-and nanoscales for cell adhesion and migration control. Int. J. Nanomed. 7, 623–630. 10.2147/IJN.S27745 22346355 PMC3277440

[B63] Torres-Costa V. Martin-Palma R. (2010). Application of nanostructured porous silicon in the field of optics. A review. J. Mater. Sci. 45, 2823–2838 10.1007/s10853-010-4251-8

[B64] Uhlir A. (1956). Electrolytic shaping of germanium and silicon. Bell Syst. Tech. J. 35, 333–347 10.1002/j.1538-7305.1956.tb02385.x

[B65] Whitehead M. A. Fan D. Mukherjee P. Akkaraju G. R. Canham L. T. Coffer J. L. (2008). High-porosity poly (ε-caprolactone)/mesoporous silicon scaffolds: calcium phosphate deposition and biological response to bone precursor cells. Tissue Eng. Part A 14, 195–206. 10.1089/ten.a.2006.0370 18333817

[B66] Williams D. F. (1999). The Williams Dictionary of Biomaterials. Liverpool: Liverpool University Press.

[B67] Williams D. F. (2008). On the mechanisms of biocompatibility. Biomaterials 29, 2941–2953. 10.1016/j.biomaterials.2008.04.023 18440630

[B68] Williams E. (2003). Forecasting material and economic flows in the global production chain for silicon. Technol. Forecast. Soc. Change 70, 341–357 10.1016/S0040-1625(02)00201-9

[B69] Wolf G. (2008). Energy regulation by the skeleton. Nutr. Rev. 66, 229–233 10.1111/j.1753-4887.2008.00027.x 18366536

[B70] Yangyang L. Fan Y. Lintao C. (2009). Osteoblast adhesion on porous silicon. Bull. Adv. Tech. Res. 3, 25–28.

[B71] Ynsa M. Dang Z. Manso-Silvan M. Song J. Azimi S. Wu J. (2014). Reprogramming hMSCs morphology with silicon/porous silicon geometric micro-patterns. Biomed. Microdevices 16, 229–236. 10.1007/s10544-013-9826-0 24305875

[B72] Zhu W. Zhang X. Wang D. Lu W. Ou Y. Han Y. (2010). Experimental study on the conduction function of nano-hydroxyapatite artificial bone. Micro Nano Lett. IET 5, 19–27. 10.3109/10731199.2012.742098 23305343

